# Tubercular appendicitis – a case report

**DOI:** 10.1186/1749-7922-1-22

**Published:** 2006-07-26

**Authors:** Sanjay Gupta, Robin Kaushik, Amanjit Kaur, Ashok Kumar Attri

**Affiliations:** 1 Department of General Surgery, Government Medical College and Hospital, Chandigarh, India; 2 Department of Pathology, Government Medical College and Hospital Chandigarh, India

## Abstract

Tuberculosis of the appendix remains a rarity despite the frequency of intestinal tuberculosis. We report a case of acute appendicitis that underwent appendectomy at our hospital, and the histopathology of the specimen revealed tuberculosis.

## Background

Although the ileocaecal region is the most commonly affected part of the intestine in intestinal tuberculosis, involvement of the appendix is rare, occurring in only about 1.5 to 3 % of cases. The appendix may either be involved secondary to ileocaecal tuberculosis, or to tuberculosis at another site within the abdomen, or, may occur in the even rarer "isolated" form, without evidence of the disease elsewhere.

Tuberculosis of the appendix presenting with the signs and symptoms of acute appendicitis is an even rarer entity. Even in areas where tuberculosis is common, it is not possible to make the correct diagnosis because the clinical picture is that of acute appendicitis, without any signs suggestive of tuberculosis infection of the organ. Therefore, the diagnosis of appendicular tuberculosis is usually made on histopathological examination of the appendectomy specimen, often received well after the patient has been discharged.

## Case report

A 12-year-old female patient presented with clinical signs and symptoms of acute appendicitis over a duration of one day. General examination revealed tenderness and rebound localised to the McBurney's point. Routine hematological and biochemical investigations were within normal limits except for raised total leucocyte count (16,000/ml). She was diagnosed as acute appendicitis and taken up for appendectomy in the emergency. Surgery was unremarkable, the appendix turgid, and showing signs of inflammation. She was discharged on the very next day.

On OPD follow up, she was detected to have wound infection that was managed conservatively. She subsequently developed an incisional hernia at the appendectomy site that was repaired electively later.

**Figure 1:**
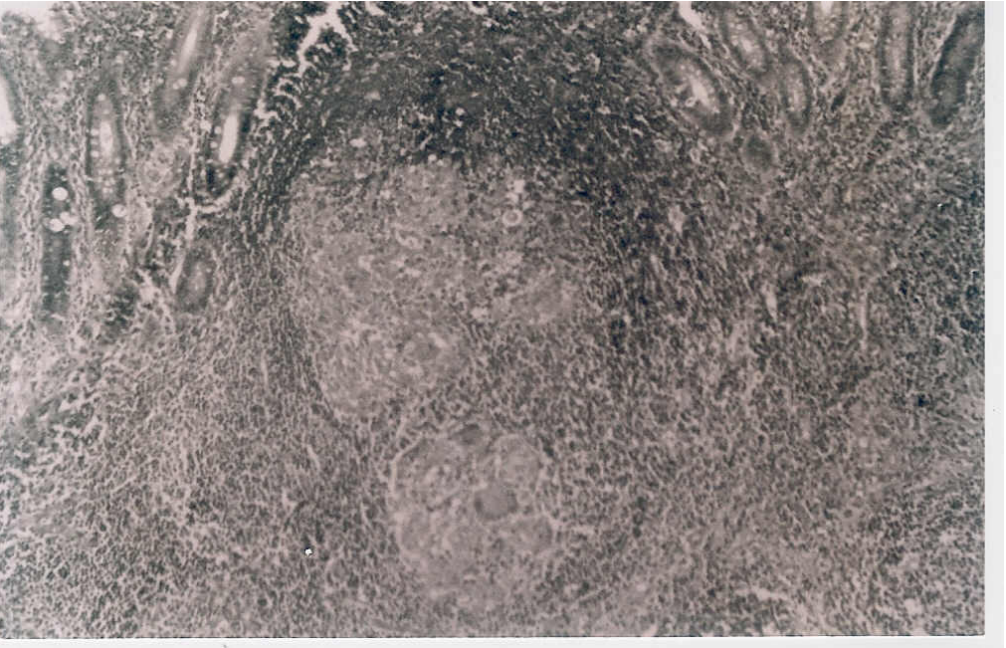
Photomicrograph showing multiple epithelioid cell granulomas in the submucosa. The overlying mucosa is focally ulcerated. (H & E × 100).

Histopathology analysis of the appendectomy specimen revealed the presence of caseating granulomas and Langhan's giant cells, suggesting tuberculosis of the appendix (Figure 1).

She was started on Anti Tubercular Therapy on OPD follow up. Efforts to detect a primary focus of tuberculosis elsewhere in the body were unsuccessful. The patient is presently well on a follow up of two and a half years.

## Discussion

Despite gastrointestinal tuberculosis being common in India, affliction of the appendix with the disease remains a rarity [1]. The reported incidence of appendicular tuberculosis in all appendectomies performed varies from 0.1 to 3.0 %, with an incidence of 1.5 to 30 % among patients who are known cases of tuberculosis [2]. Autopsy figures among patients of tuberculosis also reveal appendicular involvement in about 30 % of the cases [2]. A few authors have reported upto 46 to 70 % involvement of the appendix in patients with intestinal tuberculosis [3].

The exact mechanism of involvement of the appendix remains unclear. The various ways by which the appendix can be involved are – hematogenous, by infected intestinal contents, and, by extension of disease from neighbouring ileocaecal or genital tuberculosis [2]. A few authors consider the hematogenous route to be the common mode of spread, whereas others feel that secondary involvement of the appendix is commoner [1]. Secondary involvement of the appendix can either occur as a local extension of ileocaecal tuberculosis, as retrograde lymphatic spread from distant lesions, or as appendicular serositis and periappendicitis in peritoneal tuberculosis [1]. However, despite the ileocaecal junction being the most common site of involvement in intestinal tuberculosis, the relative infrequency of involvement of the appendix in intestinal/ileocaecal tuberculosis has been explained by the minimal contact of the luminal mucosa of the appendix with the intestinal contents [1,4]. Primary tuberculosis of the appendix has no detectable focus of infection anywhere else in the body, and is extremely rare. Ideally, to make the diagnosis of primary appendicular tuberculosis, a post mortem would be required, but for clinical purposes, this diagnosis can be made if there is an absence of any evidence of tuberculosis after thorough investigations or at laparotomy [1,2]. The mode of infection in these cases is considered to be ingestion of contaminated foods [2].

The disease can present either as a chronic disease (commonest presentation) with recurrent episodes of right iliac fossa pain, vomitings and diarrhoea, as acute appendicitis, or as a latent type that is detected incidentally [1,2]. The acute presentation occurs due to severe pyogenic infection that is superimposed on the tubercular appendix. This type of presentation is seen during the quiescent phase of pulmonary tuberculosis, if present [2]. The presence of chronic abdominal pain of long duration in young adults, pulmonary tuberculosis, poor nutritional status and loss of weight, and the presence of chronic diarrhoea have been said to be indicative of tuberculosis of the appendix [4,5], but these symptoms are of doubtful value, especially in India, where tuberculosis and amoebiasis are common [4].

As there are no pathognomic clinical features of appendicular tuberculosis, a pre-operative diagnosis is difficult. The diagnosis is usually made after histopathological examination of the appendectomy specimen. Pre-operative diagnosis does not alter the management of these patients as treatment in patients presenting with signs and symptoms of appendicitis remains appendectomy. However, anti-tubercular therapy must be started in the post-operative period if the biopsy reveals tuberculosis.

## Competing interests

The author(s) declare that they have no competing interests.

## Authors' contributions

**SG **participated in the surgery and was involved in the drafting of the manuscript.

**RK **performed the surgery, and was involved in drafting of the manuscript, and revising it critically for the intellectual content.

**A **carried out the histopathological examination and helped in drafting the manuscript.

**AKA **helped in the revision of the intellectual content and gave final approval of the version to be published.

Authors have read and approved the final manuscript.
